# Comparative insights into the gut–heart axis: cross-species and cross-population perspectives

**DOI:** 10.1080/19490976.2025.2611617

**Published:** 2026-01-11

**Authors:** Tony W.H. Tang, Kiramat Ullah, Jia-Jung Lee, Hung-Chih Chen, Patrick C.H. Hsieh

**Affiliations:** a Institute of Biomedical Sciences, Academia Sinica, Taipei, Taiwan; b Taiwan International Graduate Program in Molecular Medicine, Academia Sinica and National Yang Ming Chiao Tung University, Taipei, Taiwan; c Division of Nephrology, Department of Internal Medicine, Kaohsiung Medical University Hospital, Kaohsiung Medical University, Kaohsiung, Taiwan; d Institute of Medical Genomics and Proteomics and Institute of Clinical Medicine, National Taiwan University College of Medicine, Taipei, Taiwan; e Institute of Basic Medical Sciences, Kaohsiung Medical University, Kaohsiung, Taiwan

**Keywords:** Host-microbe interactions, cross-species models, multi-ethnic cohorts, population heterogeneity, translational microbiome research

## Abstract

Gut microbiota research has rapidly expanded our understanding of host–microbe interactions in cardiovascular diseases, yet translation of these insights remains challenged by species-specific differences and substantial population heterogeneity. In this review, we synthesize current evidence across rodents, swine, non-human primates, and multi-ethnic human cohorts to delineate conserved versus context-dependent features of the gut–heart axis. Rodent models remain indispensable for mechanistic discovery, enabling causal testing through germ-free, antibiotic-treated, and humanized microbiota platforms, whereas large-animal models better replicate human cardiac anatomy, physiology, and microbial ecology. Human studies provide essential clinical relevance, demonstrating that patients with myocardial infarction, coronary artery disease, atrial fibrillation, and heart failure harbor distinct microbial and metabolite signatures. However, these findings vary across populations due to differences in diet, lifestyle, host genetics, medication exposure, and environmental transitions. Despite taxonomic variability, several functional pathways, most notably short-chain fatty acid production, bile acid biotransformation, and aromatic amino acid metabolism generating molecules such as trimethylamine-*N*-oxide and phenylacetylglutamine, consistently associate with cardiovascular risk. At the same time, population-specific features, including glycan–microbe interactions shaped by ABO and FUT2 genotypes, diet-responsive metabolite profiles, and variable drug–microbiome interactions, highlight the importance of genetic and environmental context. By integrating cross-species and cross-population evidence, this review outlines a framework for identifying robust microbial pathways, clarifying their translational boundaries, and guiding the development of microbiota-informed diagnostics and interventions that account for biological, cultural, and environmental diversity.

## 1 Introduction

### 1.1 The gut–heart axis beyond single models and populations

Cardiovascular diseases are increasingly recognized as disorders shaped not only by host genetics and lifestyle but also by the intestinal microbiota.^1^^,^^2^ Yet, translating microbiome discoveries into cardiovascular medicine remains challenging. One major barrier is species-specific differences in microbiota composition and host physiology that complicate extrapolation from animal models to humans.^3‐5^ Another is population-level heterogeneity, where cultural, dietary, and environmental contexts shape microbial communities and cardiovascular risk.^6‐8^

Among the best-characterized microbial metabolites, short-chain fatty acids (SCFAs)^4^ and trimethylamine-*N*-oxide (TMAO)^5^^,^^6^ consistently emerge across studies as key mediators of cardiovascular outcomes.^9‐12^ SCFAs are generally cardioprotective,^13^^,^^14^ whereas TMAO is linked to adverse risk;^15^^,^^16^ yet their effects vary across species and populations due to differences in physiology, diet, and microbial composition, underscoring the need for cross-species and cross-population frameworks to enable translation.^17‐19^

### 1.2 Limitations of single-species models

Preclinical studies have been indispensable for dissecting microbiota–cardiovascular interactions, yet their translational value is constrained by species-specific differences. A fundamental trade-off exists: animal models provide tractable systems for discovery but cannot fully capture the ecological and physiological complexity of humans. In parallel, reductionist approaches, including germ-free or antibiotic-treated mice and minimal synthetic consortia, remain essential for probing causality,^10,20‐22^ yet they oversimplify the gut ecosystem, lacking community-level diversity, cross-feeding, and resilience. These contrasts underscore that no single model suffices; advancing translation requires integrating insights from reductionist systems with comparative evidence across multiple species and human cohorts.^15,16,22-24^

### 1.3 Limitations of single-population studies

Most microbiome studies are conducted in genetically and environmentally homogeneous cohorts, which constrains discovery of gene-microbe interactions and limits generalizability to other populations. ^21,23–25^ Additional variability arises from medications, comorbidities, and lifestyle exposures that are often incompletely controlled, making it difficult to disentangle primary microbial signals from secondary influences. Single time-point designs further capture only static snapshots, overlooking the dynamic fluctuations of microbiota and metabolites that may parallel cardiovascular trajectories.^25^ Rather than treating such heterogeneity as noise, comparative analyzes across multi-ethnic and longitudinal cohorts provide opportunities to distinguish universal microbial signatures from population-specific mechanisms. These considerations emphasize the importance of integrating diversity, longitudinal monitoring, and careful clinical annotation to advance precision microbiome-cardiovascular medicine.

### 1.4 Scope and objectives of this review

This review aims to provide a critical evaluation of the gut–heart axis by integrating insights from both cross-species and cross-population perspectives. Specifically, we synthesize evidence from diverse human populations to highlight how population context, including genetic and environmental factors, shapes microbiota-cardiac associations. In parallel, we compare findings across animal models to identify conserved microbial pathways as well as species-specific constraints that influence translational relevance.

By distinguishing universal mechanisms from context-dependent differences, this comparative framework seeks to refine translational strategies, clarify why some microbiome-targeted interventions succeed in humans while others fall short, and accelerate the development of microbiota-informed diagnostics and therapeutics. Ultimately, our goal is to provide more precise guidance for future research design, enabling a nuanced and clinically relevant understanding of microbiome–cardiovascular interactions that can inform precision medicine.

## 2 Cross-species analysis: Animal models of the gut–heart axis

**Figure 1.**
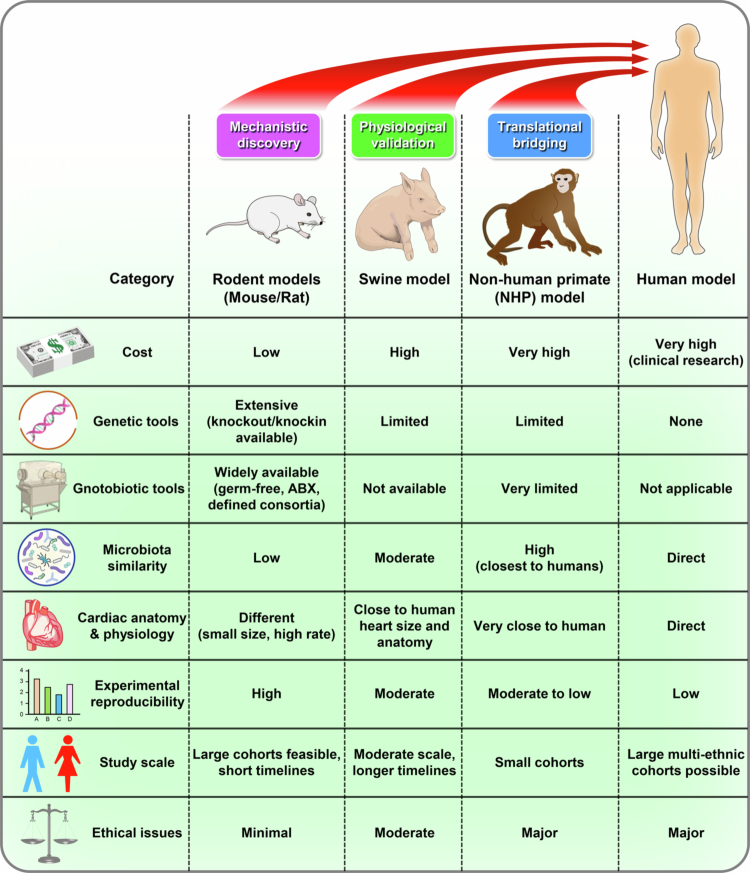
Cross-species comparative framework for studying the gut–heart axis. Comparison of rodent, swine, non-human primate, and human models in cardiovascular microbiome research. Rodents enable genetic manipulation and mechanistic discovery but differ markedly from humans in microbiota and physiology. Swine provide human-like anatomy and physiology yet are costly and lack gnotobiotic tools. Non-human primates most closely resemble human microbiota and cardiovascular features but face ethical and logistical barriers. Human studies offer direct clinical relevance and multi-ethnic diversity, though limited by heterogeneity and the paucity of causal, long-term trials. Together, these models serve complementary roles in mechanistic discovery, physiological validation, translational bridging, and clinical application.

Animal models provide indispensable platforms for dissecting microbiota–cardiovascular interactions, offering mechanistic resolution not feasible in human cohorts. No single species fully recapitulates human microbiota and physiology, but each contributes complementary strengths: rodents enable causal testing, swine approximate human cardiac anatomy and metabolism, and non-human primates most closely mirror human immune and microbial features. Human studies anchor these findings in clinical reality but face heterogeneity and confounding. Together, these approaches form a continuum that clarifies both conserved pathways and species-specific constraints in the gut-heart axis (Figure 1). The following subsections detail each model’s advantages, limitations, and translational implications.

### 2.1 Rodent models (Mouse/Rat)

Rodents, particularly mice and rats, represent the most widely used animal models in gut microbiota-cardiac research. Their popularity stems from several advantages: genetic tractability, relatively low maintenance costs, and the availability of well-established cardiovascular disease models, including myocardial infarction (MI), heart failure with preserved ejection fraction (HFpEF), and transverse aortic constriction.^13^^,^^21^ Moreover, rodent colonies allow for stringent control of diet, housing, environment, and medication, thereby reducing confounding variables and enabling reproducible mechanistic studies.^10^^,^^22^

Germ-free, antibiotic-treated, and humanized microbiota–associated rodent models enable direct interrogation of microbiota–host interactions and allow causal links to cardiac pathophysiology to be established.^13^^,^^21^^,^^26^ Loss of commensal microbes impairs post-MI repair by disrupting immune homeostasis,^21^ whereas supplementation with microbial metabolites such as acetate or propionate improves myocardial adaptation to pressure overload.^13^ Enrichment of butyrate-producing taxa after MI similarly reduces inflammation and supports favorable remodeling, suggesting a cardioprotective role for microbial butyrate.^14^ Microbial metabolites also modulate host gene expression through epigenetic mechanisms, providing an additional layer of microbiota-derived regulation.^11^ To enhance reproducibility, defined consortia such as the GM15 synthetic microbiota offer standardized communities for mechanistic and translational studies.^27^ Collectively, these findings highlight the value of rodent systems in uncovering microbiota-derived metabolites and signaling pathways relevant to cardiovascular disease.

Nevertheless, important limitations remain. Rodent gut communities differ markedly from humans, with disproportionately high levels of *Lactobacillus, Muribaculaceae*, and segmented filamentous bacteria rarely seen in human microbiota.^22^ This divergence constrains direct translational inference. Moreover, cardiovascular experiments frequently use acute injury models such as surgical MI or transverse aortic constriction, which rapidly alter the gut microbiome via stress, inflammation, and shifts in intestinal motility.^21^^,^^28^ These abrupt perturbations raise concerns about reverse causality, as many microbial changes may reflect consequences of cardiac injury rather than upstream drivers of disease.^21^

Furthermore, the routine use of antibiotic conditioning in humanized or gnotobiotic mouse studies disrupts native microbial communities, alters metabolite pools, and resets immune baselines, introducing significant confounding when interpreting microbiota-cardiac interactions.^26^^,^^28^ Rodents also differ from humans in metabolic rate, immune phenotypes, and cardiovascular physiology, factors that shape disease trajectories and therapeutic responses. In addition, current murine models inadequately capture the chronic, progressive remodeling that characterizes human atherosclerosis and heart failure, limiting their ability to model long-term host–microbiome co-adaptation.^12^ While indispensable for mechanistic discovery, these constraints underscore the need for cautious interpretation and complementary validation in large-animal or humanized systems that more closely mirror human microbial ecology and disease chronicity.

Most rodent studies rely on inbred strains, limiting the genetic diversity relevant to human populations. Outbred models such as Diversity Outbred (DO) and Collaborative Cross (CC) mice better capture heterogeneity in immune responses, metabolic traits, and microbiota composition. DO mice exhibit substantial inter-individual variation in microbial communities and metabolite profiles, enabling detection of gene–microbiota interactions that are undetectable in homogeneous strains.^29^ CC lines similarly facilitate mapping of host genetic variants that influence microbial colonization, mucosal glycosylation, and cardiometabolic phenotypes.^30^ Using these genetically diverse resources provides a more realistic platform for modeling population-level variation in microbiota–cardiac interactions and enhances the translational relevance of preclinical studies.

These findings highlight a persistent translational gap between murine and human studies. Many microbial signatures identified in large human cohorts cannot be reproduced in rodents due to substantial compositional and functional divergence.^31^ Rodents also differ from humans in metabolic rate, immune regulation, and cardiovascular physiology, factors that influence disease progression and therapeutic response. Although invaluable for mechanistic discovery, murine models of atherosclerosis and heart failure possess inherent constraints, requiring caution when extrapolating results across species.^12^

### 2.2 Swine models

Swine represent an important large-animal platform for interrogating the gut–heart axis because their cardiac size, coronary anatomy, heart rate, and metabolic features more closely resemble those of humans than rodents. These similarities make pigs particularly suitable for modeling MI, ischemia/reperfusion injury, and post-infarction remodeling under controlled dietary and environmental conditions, thereby improving translational relevance.

Recent studies highlight their utility, in which butyrate supplementation promotes cardiac output and vasorelaxation, supporting the translational application of using microbiota-derived SCFAs to modulate cardiovascular function.^15^ Comparative microbiome profiling in Shaziling pigs revealed enrichment of *Prevotella* and *Treponema*, consistent with their high-fiber diets, together with breed-specific taxa such as *Blautia*, *Clostridium* clusters, *Lactobacillus johnsonii*, *Lactobacillus amylovorus*, and *Clostridium butyricum*.^32^ These features distinguish porcine communities from humans and highlight their value for studying how diet-microbiota interactions influence host lipid metabolism.^32^

Nevertheless, important limitations remain. Unlike rodents, gnotobiotic or germ-free pig models are not widely available, restricting the ability to perform direct mechanistic studies of microbiota causality. Furthermore, swine studies require substantial investment in housing, specialized facilities, and extended experimental timelines. Despite these challenges, swine models serve as an important intermediate platform bridging rodent discoveries and human clinical translation. Their physiological similarity to humans enables validation of candidate microbial pathways and therapeutic strategies, while ongoing dietary modulation and metabolite supplementation studies illustrate their potential in modulating human pathophysiology more faithfully than rodent systems.^15^^,^^32^

### 2.3 Non-human primate models

Non-human primates (NHPs) offer a unique translational bridge between rodent studies and human clinical research in the gut-heart axis. Compared to rodents, NHPs share greater similarity to humans in gut microbial composition, immune system architecture, and cardiovascular physiology, thereby providing a more faithful platform for modeling microbiota-host interactions relevant to cardiovascular diseases. Comparative analyzes have shown that macaque gut microbiota more closely resembles that of humans, with higher abundances of *Bacteroides*, *Prevotella*, and *Faecalibacterium* relative to rodent.^33^ Macaque studies further reveal overlapping strains within *Ruminococcaceae* and *Lachnospiraceae* that are shared with humans, although primate-specific clades persist, shaped by distinct diets and environments.^34^ These similarities extend to both taxonomic diversity and functional pathways, underscoring the value of NHPs as superior models for translational microbiome research. Moreover, inter-individual and inter-population variation in NHP microbiomes parallels that seen in humans, supporting their utility in capturing ecological and evolutionary dimensions of host-microbiota interactions.^18^^,^^34^ For instance, Mediterranean diet-fed female macaques exhibited significant higher abundance of *Lactobacillus* in their mammary gland microbiota than their Western diet-fed.^35^

Recent studies highlight the feasibility of applying NHP models in cardiac research. In an ischemia/reperfusion model with immunosuppression, *Chen et al.* showed that immunosuppression alters gut microbiota, and that these microbial changes modulate the therapeutic efficacy of cell-based cardiac therapy.^16^ Cross-species analyzes further revealed that butyrate-producing bacteria such as *Butyricimonas virosa* are enriched during recovery after IR injury in both humans and NHPs, pointing to conserved microbiota-mediated protective mechanisms across species.^14^ Beyond descriptive comparisons, NHP ischemia/reperfusion models offer unique advantages over human ST-segment elevation myocardial infarction (STEMI) studies, including controlled diets, standardized environments, and precise regulation of the timing and extent of myocardial injury,^16^ thereby reducing confounding variability present in clinical cohorts. These features provide opportunities to interrogate microbial contributions to cardiovascular repair within a clinically relevant yet experimentally tractable system.

Despite these strengths, NHP models face persistent challenges, including substantial ethical considerations, high financial costs, and the logistical complexity of long-term studies. The absence of gnotobiotic or germ-free NHPs also limits direct causal testing of microbiota–host interactions. Nonetheless, because NHPs recapitulate human-like microbiota and cardiovascular physiology, they remain indispensable for validating candidate microbial pathways and therapeutic strategies identified in rodent models prior to clinical translation.

### 2.4 Human studies

Human studies provide essential clinical evidence linking the gut microbiota to cardiovascular disease. Large cohort and case–control analyzes consistently show that patients with myocardial infarction, coronary artery disease, atrial fibrillation, and heart failure exhibit distinct gut microbial profiles, marked by reduced SCFA-producing taxa and enrichment of pathobionts. These alterations often coincide with elevated circulating metabolites such as TMAO, which predict adverse cardiovascular outcomes.^6^^,^^18^

Beyond cross-sectional associations, prospective studies show that several microbiota-related metabolites, including TMAO,^17^ phenylacetylglutamine,^36^ and circulating microbial DNA,^37^ predict future cardiovascular events independent of traditional risk factors. Multi-center cohorts across Europe, North America, and Asia further reveal both shared and population-specific microbial signatures shaped by ethnicity, diet, and lifestyle.^18^ Loss of SCFA-producing bacteria is a consistent feature of chronic heart failure, aligning with experimental evidence that depletion of commensals impairs post-MI repair and that butyrate-producing microbes exert cardioprotective effects.^14^^,^^21^

Beyond compositional changes, integrative multi-omics approaches have revealed key mechanistic links between the microbiome and cardiovascular risk. Circulating microbial metabolites are associated with adverse outcomes and, in several prospective cohorts, predict incident coronary artery disease, recurrent MI, and heart failure hospitalization.^6^^,^^17^^,^^21^^,^^36^^,^^38^^,^^39^ Moreover, fecal microbiota transplantation from patients with cardiovascular disease into germ-free mice reproduces aspects of the disease phenotype, providing causal support for microbiota–host interactions.^13^^,^^21^

Despite these advances, important limitations persist. Human microbiome studies are confounded by substantial inter-individual variation driven by diet, medications, comorbidities, and host genetics, complicating efforts to identify universal microbial markers. Variability in sample type (stool vs. mucosal biopsy) and study design (cross-sectional vs. longitudinal) further restricts comparability across cohorts. Moreover, although early trials of probiotics, prebiotics, and fecal microbiota transplantation suggest potential benefit, adequately powered randomized controlled studies with long-term follow-up remain scarce.^12^

Taken together, human studies are essential for defining the microbiota’s contribution to cardiovascular disease, providing both epidemiological and mechanistic insight. They also highlight the need for standardized methodologies, diverse population cohorts, and well-controlled interventional trials to advance microbiome-informed cardiovascular medicine. To integrate evidence across experimental systems, Table 1 summarizes key cardiometabolic microbiome features identified in rodent, swine, non-human primate, and human studies.

**Table 1.** Cross-species summary of gut microbiota evidence in cardiovascular disease.

Model/Species	Key microbial features	Representative findings	Major strengths	Limitations	References
Rodents (Mouse/Rat)	Dominated by *Lactobacillus*, *Muribaculaceae*; high divergence from human microbiota	SCFAs (acetate/propionate) improve pressure-overload adaptation; GF/ABX models confirm causality; butyrate-producers protect post-MI	Causality, genetics, gnotobiotic tools	Microbiota differs from humans; acute injury models; altered metabolism	[13,14,21,22,26‐30]
Swine	Diet-driven *Prevotella*, *Treponema*; partial overlap with human strains	Butyrate increases cardiac output & vasorelaxation; diet–microbiome–lipid interactions	Human-like cardiac physiology; translational validation	Limited gnotobiotic tools; high cost	[15,32]
Non-Human Primates	Closest to humans in microbial taxa (*Bacteroides, Prevotella, Faecalibacterium*) and immune–CV physiology	Microbiota shifts modify cardiac cell therapy response; conserved post-IR enrichment of butyrate-producers	Highest phylogenetic similarity; controlled diet & environment	Ethical & cost barriers; no gnotobiotic systems	[16,33,34,35]
Humans	Ethnicity-, diet-, and geography-dependent microbial structure	TMAO predicts incident CVD; PAGln predicts HF outcomes; microbial cfDNA predicts long-term MACE	Clinical relevance; multi-ethnic evidence	Confounding factors; heterogeneous designs	[6,17,36‐39,47]
Conserved Pathways	SCFA–GPCR signaling; LPS–TLR4 inflammation; aromatic amino acid metabolites (PAGln, IS); HDAC inhibition	SCFAs protective across species; LPS drives remodeling; PAGln linked to HF; cfDNA predicts events	Mechanistic convergence	Species differences in bile acid pools & metabolic rates	[1,2,3,11,40,41]

A cross-species overview of cardiovascular microbiome findings, summarizing key microbial features, representative results, and model-specific strengths and limitations across rodents, swine, non-human primates, and humans.

### 2.5 Cross-species conserved and species-specific microbial pathways

Multiple microbiota-derived pathways contribute to cardiovascular regulation across species. SCFAs act through GPCR-mediated anti-inflammatory and metabolic effects, while aromatic amino acid metabolites such as TMAO, PAGln, indoxyl sulfate, and phenylacetate modulate platelet activation, adrenergic signaling, and vascular inflammation. Several of these metabolites predict incident cardiovascular events in prospective human cohorts, underscoring their translational relevance.^40^

Bile acid metabolism represents another conserved axis: microbiota-driven FXR and TGR5 signaling regulates lipid handling, glucose homeostasis, and vascular tone in humans, swine, and non-human primates, although rodents possess distinct bile acid intermediates that limit direct extrapolation. Innate immune pathways, including LPS–TLR4 and other microbe-associated molecular patterns, constitute a broadly conserved inflammatory mechanism contributing to adverse cardiac remodeling.^1‐3^

Microbial metabolites further modulate host epigenetic landscapes through HDAC inhibition, a broadly conserved mechanism linking gut ecology to cardiometabolic regulation.^11^ Population-enriched metabolites such as trimethyl-5-aminovaleric acid (TMAVA) illustrate how microbe-host interactions can differ across cohorts yet remain mechanistically active in rodent validation studies.^41^ Emerging evidence also highlights circulating microbial DNA as a marker of barrier dysfunction and systemic inflammation, predicting long-term outcomes in human MI and paralleling post-injury microbial translocation in animal models.^37^ Together, these pathways demonstrate that while SCFAs remain a key conserved signaling axis, a multi-pathway framework is required to capture both shared and context-dependent microbiota–cardiac mechanisms across species.

## 3 Cross-population variations in the human gut–heart axis

**Figure 2.**
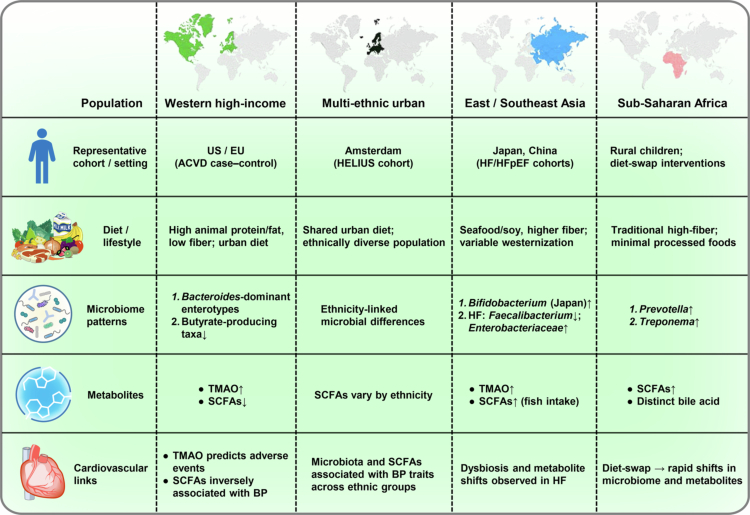
Cross-population variations in the human gut–heart axis. Representative cohorts illustrate how ethnicity, geography, diet, and lifestyle shape microbiota–cardiovascular associations. Human cohorts across Western, Asian, African, multi-ethnic urban, and disease-specific populations reveal that microbiota composition and metabolites vary with diet, ethnicity, and environment. While SCFA-producing taxa are associated with favorable cardiovascular outcomes and TMAO is associated with increased cardiovascular risk, the specific microbial signatures differ by population, underscoring the need for population-aware translational strategies.

Human studies establish the clinical relevance of the gut–heart axis but are strongly influenced by population heterogeneity. Geography, culture, and host genetics shape microbial composition and metabolite output, producing both shared and population-specific cardiovascular associations. Table 2 summarizes key multi-ethnic cohorts, including study design and cardiovascular outcomes, to contextualize this variation. Comparative analysis across diverse, and when available, longitudinal, cohorts helps distinguish conserved pathways from context-dependent effects and informs precision-targeted interventions (Figure 2). The following subsections outline representative examples of cross-population variation and their cardiovascular implications.

**Table 2.** Summary of key human cohort studies examining gut microbiome and cardiovascular outcomes.

Study/population	Reference	Study design	Sample size	Microbiome features	Metabolites	Key findings	Longitudinal evidence
MESA (USA)	【Ref 17】	Prospective cohort	~5,000	Not primary microbiome, but metabolite-focused	TMAO	Higher TMAO predicts incident CVD independent of traditional risk factors	Incident CVD
Cleveland Clinic cohorts	【Ref 1,2】	Case-control + prospective	1,876	Gut bacteria associated with TMAO pathway	TMAO	TMAO elevated in CAD & predicts 3-year mortality	Prospective mortality
PAGln & HF Study (USA)	【Ref 36】	Prospective cohort	1,162	N/A	PAGln	PAGln predicts HF hospitalization and mortality	HF outcomes
Circulating Microbial DNA in STEMI (Taiwan)	【Ref 37】	Prospective	215	Translocated microbial DNA	Microbial cfDNA	Higher microbial DNA predicts 3-year MACE	Longitudinal
PREDIMED Trial (Spain)	【Ref 49】	Randomized dietary intervention	7,216	Microbial metabolite pathways	Choline → TMAO	TMAO-related metabolites predict future CAD	Incident CAD
HCHS/SOL (Hispanic/Latino)	【Ref 47】	Prospective, multi-ethnic	16,400	Microbiome-associated dietary patterns	SCFA-related metabolites	Diet–microbiome axis predicts metabolic risk	Prospective
Chinese CAD cohorts	【Ref 31】	Case–control	405	Dysbiosis signatures	TMAO, SCFA	Association with CAD but no temporal inference	Cross-sectional

Human studies vary widely in evidentiary strength. Cross-sectional designs identify associations, whereas prospective cohorts and interventional trials provide predictive or causal inference. Longitudinal evidence linking microbiota-derived metabolites (e.g., TMAO, PAGln) to incident cardiovascular events is robust, while repeated microbiome sampling remains a critical gap across cohorts.

### 3.1 Baseline microbiota differences by ethnicity and geography

Gut microbiota composition varies substantially across populations due to ethnicity, geography, diet, and environment. Western cohorts often show Bacteroides-dominated enterotypes, whereas many Asian populations consuming plant-rich diets exhibit higher *Prevotella* abundance*.*^42‐44^ These differences extend to functional pathways, including amino acid and lipid metabolism, highlighting deep cultural and environmental influences on baseline microbial ecology.^45^

Diet and lifestyle strongly shape microbial stability. Westernized living, characterized by high-fat diets, antibiotic exposure, and urbanization, is linked to reduced SCFA-producing taxa such as *Faecalibacterium prausnitzii* and *Bifidobacterium*.^23^^,^^46^^,^^47^ Conversely, traditional high-fiber Asian diets promote greater fermentative capacity and elevated SCFA levels. A multi-omics study of East Asian and White participants living in the same U.S. region found persistent differences in Bacteroidetes and *Akkermansia muciniphila* abundance even after dietary adjustment, suggesting an important role for intrinsic host–microbiota interactions.^48^

Geography and migration also remodel the microbiome. Comparative studies of atherosclerosis in Sweden and China identified both shared and region-specific microbial depletions, reflecting local strain-level adaptations.^48^ In the multi-ethnic HELIUS cohort, cardiovascular associations of taxa differed by ancestry: *Akkermansia muciniphila* associated with favorable risk in African-Surinamese individuals, whereas *Ruminococcaceae* members were protective in Moroccan participants.^47^ Migration-driven dietary acculturation, such as reduced fiber intake among U.S. Hispanic/Latino adults, leads to decreased *Prevotella* and *Clostridia*, accompanied by higher cardiometabolic risk.^49^ Similar trends occur in Asian immigrants.^49^^,^^50^

Across populations, focusing solely on species-level variation risks overlooking broader ecological principles. Functional redundancy allows distinct taxa to perform similar roles, such as SCFA production, bile acid biotransformation, branched-chain amino acid degradation, or mucin glycan utilization, maintaining metabolic stability despite taxonomic differences.^45^^,^^47^^,^^48^ For example, although *F. prausnitzii* is abundant in many Western cohorts, its butyrogenic function can be provided by *Roseburia*, *Eubacterium rectale*, or *Butyricicoccus* in Asian or African populations.^45^^,^^47^

Within this framework, a “healthy microbiome” is best defined by functional capacity, stable SCFA production, preserved mucosal integrity, immunoregulatory metabolite output, and resilience to environmental shifts, rather than by a fixed set of species. Multi-ethnic studies support this view: while taxonomic profiles vary markedly across populations, core functional modules such as carbohydrate fermentation, amino acid catabolism, and bile acid metabolism consistently associate with favorable cardiometabolic traits.

Overall, integrating functional redundancy with pathway-level conservation provides a robust, population-aware framework for interpreting gut–heart associations while minimizing overinterpretation of taxonomic variability.

### 3.2 Population-specific findings in cardiac disease

Cardiac diseases such as MI, coronary artery disease, and chronic heart failure are consistently linked to gut microbiota dysbiosis, though the specific alterations vary across populations. In chronic heart failure, patients typically show a depletion of SCFA-producing taxa (e.g., *Ruminococcaceae, Lachnospiraceae*) alongside an enrichment of *Proteobacteria* and *Enterococcus*, leading to reduced microbial diversity and impaired metabolic resilience.^21^ These changes are often paralleled by shifts in metabolite production, including elevated lactate and reduced SCFA levels, thereby compromising host–microbiota homeostasis.

Population-based metabolomic studies further reveal distinct ethnic and regional signatures. In U.S. and European cohorts, phenylacetylglutamine has been strongly linked to adverse heart failure outcomes via adrenergic receptor signaling.^36^^,^^40^ In contrast, in Chinese cohorts, *N*-trimethyl-5-aminovaleric acid, a lysine-derived microbial metabolite, has been implicated in cardiac hypertrophy and maladaptive remodeling.^41^ Similarly, in acute coronary syndromes, microbiota–metabolite biomarker panels have distinguished MI from stable coronary artery disease, with taxa such as *Streptococcus*, *Alistipes*, and *Lactobacillus* emerging as discriminant markers.^31^

Intervention and longitudinal evidence with diet-centric strategies, including Mediterranean and Green-Mediterranean patterns, show microbiome remodeling, enhanced SCFA pathways, and improvements in cardiometabolic biomarkers.^44^^,^^45^ In a cohort of acute MI patients, enrichment of *Streptococcus salivarius* and *Klebsiella pneumoniae*, together with depletion of *Roseburia hominis*, predicted major adverse cardiovascular events during long-term follow-up.^31^ Circulating microbial DNA signatures (e.g., *Corynebacterium, Staphylococcus aureus*) were also correlated with poor prognosis in STEMI patients, raising the possibility that systemic microbial dissemination may contribute to disease severity.^37^

Comparative studies highlight cross-cohort variability. In Chinese patients with atherosclerosis, depletion of *Roseburia inulinivorans* and *Eubacterium eligens* distinguished cases from controls, while validation in Swedish cohorts revealed both overlapping and distinct strain-level adaptations.^48^^,^^51^ Multi-ethnic metabolomics analyzes identified 73 microbiota-related metabolites associated with incident coronary artery disease, including metabolites associated with lower risk, such as taurine and glycine, and risk-associated metabolites such as sphingomyelin and *N*-acetylglutamate, findings validated across Black, White, and Chinese populations.^38^^,^^49^

Collectively, these studies highlight that population-specific microbial and metabolic signatures not only influence cardiovascular disease risk but also shape prognosis and response to therapy. Recognizing these variations is crucial for advancing toward personalized, context-aware microbiome-based diagnostics and interventions in cardiovascular medicine.

### 3.3 Host genetics, lifestyle, and environmental factors

Intrinsic host–microbiota interactions, shaped by genetic and physiological factors, regulate microbial colonization and metabolic function. Host-controlled features, such as mucosal glycan composition, epithelial metabolism, barrier integrity, and immune-mediated microbial containment, create individualized ecological niches that drive inter-individual variation in gut communities. These intrinsic processes are further influenced by population-level genetic diversity. Variants affecting mucin glycosylation, epithelial transporters, innate immune receptors, and xenobiotic metabolism differ across ethnic groups and alter microbial colonization and metabolite output.^19^^,^^42^

Beyond ethnicity and geography, host genetics, diet, and environmental exposures exert profound influence on gut–heart interactions. Multi-omics studies demonstrate that these factors collectively explain a substantial proportion of variability in plasma metabolomes, with diet acting as the dominant driver.^45^ Host genetic factors modulate microbial pathways independent of diet. Variants in ABO and FUT2 alter mucosal glycosylation and the availability of *N*-acetylgalactosamine, shaping colonization and metabolic capacity of saccharolytic commensals such as *Faecalibacterium prausnitzii*.^19^^,^^42^ These glycan–microbe interactions influence SCFA production and downstream cardiometabolic traits. Twin studies further demonstrate that microbiota-derived metabolites, including sphingomyelins and glycerophospholipids, affect lipid phenotypes even among genetically similar individuals, underscoring the interdependence of heritable and microbial factors.^34^

Dietary habits act as powerful environmental modulators of these genetically influenced pathways. Controlled interventions, including Mediterranean, Green-Mediterranean, and Microbiome Enhancer Diet trials, show that dietary shifts reshape microbial composition, enhance SCFA production, and activate branched-chain amino acid degradation pathways, leading to improved cardiometabolic biomarkers.^43‐45^ Prospective cohorts further demonstrate diet-associated metabolite patterns, such as higher TMAO with animal-based diets and higher enterolactone with plant-based diets, that predict cardiovascular outcomes.^39^

Complementing these genetic influences, intrinsic host physiology also regulates microbial function. Epithelial oxygen gradients and metabolic programming determine microbial fermentation profiles, while immune–microbe interactions, such as IgA-mediated spatial segregation and antigen sampling, maintain ecological stability. These genetically and immunologically encoded mechanisms vary across populations, providing mechanistic explanations for ethnic- and region-specific differences observed in multi-ethnic cohorts.^34^

Pharmacological exposures strongly influence gut microbial ecology and help explain inter-individual and inter-population variability. In experimental models, non-absorbable antibiotics lower aromatic amino acid metabolites and lessen myocardial infarction severity, whereas antibiotic-induced dysbiosis elevates TMAO and accelerates atherosclerosis.^28^^,^^40^ Cardiovascular drugs such as statins also interact bidirectionally with the microbiome: statins alter bile acid pools and shift taxa in a genotype-dependent manner, while microbial enzymes modulate drug metabolism and bioavailability.^19^^,^^22^^,^^40^^,^^42^ Lifestyle transitions associated with urbanization and dietary acculturation similarly reduce fiber-degrading taxa and track with higher cardiometabolic risk.^49^^,^^50^ Incorporating these drug– and lifestyle–microbiome interactions will be essential for population-informed cardiovascular therapeutics.

Although intrinsic host factors shape microbial colonization, many interactions remain modifiable through diet, prebiotics, and environmental inputs, as shown in humanized mouse and non-human primate models.^16^^,^^26^^,^^43^^,^^45^ Genetics, diet, medications, and environment dynamically interact with the microbiome to influence host metabolism and cardiovascular phenotypes. Recognizing this plasticity is essential for developing precision microbiome-based interventions tailored to individual biological and environmental contexts.

## 4 Future perspective

### 4.1 Develop harmonized, translationally relevant animal models

**Figure 3.**
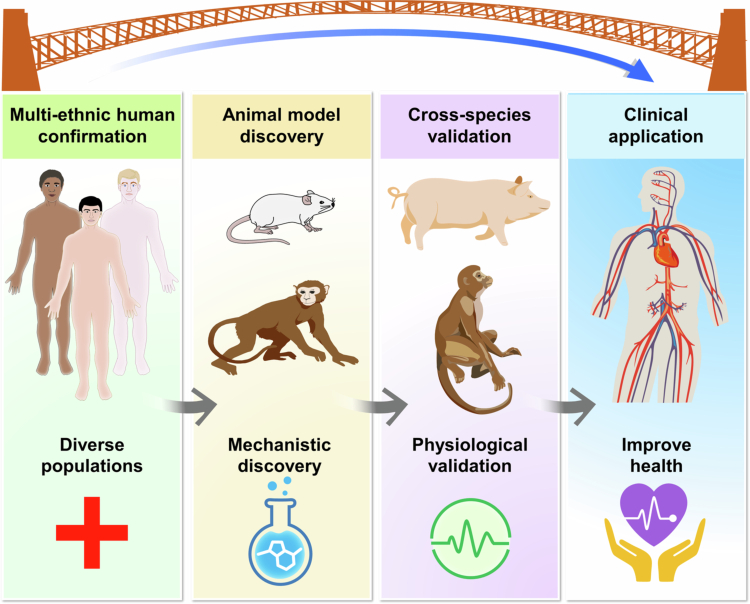
Comparative translational framework for microbiome–cardiovascular research. Schematic illustration of an integrative framework linking evidence across models and populations. Multi-ethnic human cohorts identify microbial pathways and metabolites associated with disease. These human-derived findings are then explored in rodents, swine, and non-human primates to delineate mechanisms and clarify host-specific versus conserved microbiome-cardiac interactions. Cross-species validation refines causal inference and guides the development of targeted interventions. Insights from clinical translation, in turn, generate new hypotheses that feed back into diverse human populations, completing a continuous cycle of discovery and validation.

To strengthen translational relevance, the workflow bridging microbiome discoveries and cardiovascular applications is best conceptualized as a circular, bidirectional framework rather than a one-directional sequence (Figure 3). Multi-ethnic human studies provide the starting point for identifying microbial signatures, ecological patterns, and metabolite pathways linked to disease. These population-derived findings then undergo mechanistic investigation in rodents and non-human primates, followed by cross-species physiological validation in models such as swine that better approximate human anatomy and cardiac physiology. Importantly, results from these preclinical systems do not simply confirm human findings, they often refine, redirect, or challenge original hypotheses, emphasizing the iterative nature of this process. The insights gained subsequently inform clinical interventions aimed at modifying microbiota-derived metabolites, dietary exposures, or host-microbe interactions. Outcomes from these clinical applications then feed back into population studies, establishing a continuous feedback loop that improves reproducibility, enhances causal inference, and identifies microbial pathways with genuine translational potential across species and populations.

### 4.2 Design multi-ethnic cardiovascular microbiome clinical trials

Most current clinical evidence comes from single-population cohorts, which limits generalizability. To advance toward precision medicine, there is an urgent need for multi-ethnic, longitudinal, and large-scale cardiovascular microbiome trials. Such studies should adopt standardized metagenomic, metabolomic, and proteomic pipelines to enable cross-cohort comparability. Crucially, they must be designed to capture gene–diet–microbiome interactions across diverse populations, accounting for ethnicity-specific dietary habits, host genetics, and environmental exposures. Building multi-ethnic consortia and global collaborations will be key to identifying both universal microbial signatures and population-specific risk factors, ultimately enabling the development of culturally tailored preventive and therapeutic strategies. Future multi-ethnic cardiovascular microbiome trials should incorporate repeated microbiome measurements to capture individual trajectories and relate within-person microbial dynamics to future disease progression.

### 4.3 Targeted microbiota-based therapeutics for specific populations and disease phenotypes

The next frontier lies in targeted microbiota-based therapeutics, moving beyond general probiotic or prebiotic interventions toward strategies tailored to disease context and population background. Examples include supplementation with butyrate-producing taxa or SCFA analogs for post-MI remodeling, inhibition of TMAO-producing pathways for atherosclerosis, and precision probiotics for coronary artery disease or heart failure patients with distinct microbial deficits. Advances in engineered probiotics, postbiotics, and microbial metabolite modulators provide promising avenues for therapy. Crucially, these interventions must be validated in population-aware clinical studies to ensure both efficacy and safety, particularly given ethnic and environmental differences in microbiome composition and host response.

## 5 Conclusion

The study of the gut–heart axis has uncovered profound connections between intestinal microbiota, microbial metabolites, and cardiovascular diseases. However, meaningful clinical translation requires perspectives that span both species and populations. Studies in rodent, swine, and non-human primate models provide indispensable mechanistic and physiological knowledge, yet their inherent limitations highlight the need for careful integration with human data. Likewise, human studies reveal strong associations between gut microbiota and cardiovascular outcomes, but these findings are shaped by genetic, cultural, and environmental diversity, underscoring the necessity of population-aware interpretation. Importantly, across species and cohorts, SCFAs are consistently associated with favorable cardiovascular profiles, whereas TMAO signals risk, illustrating conserved yet context-dependent microbial pathways.

Future progress will depend on experimental frameworks that explicitly account for species-specific and ethnicity-specific features, thereby reflecting the ecological and physiological complexity of microbiome–cardiovascular interactions. Coordinated multi-species and multi-ethnic collaborations will be critical to advancing robust microbiota-based diagnostics and therapeutics for cardiovascular diseases.
